# Norman Bleehen

**DOI:** 10.1038/sj.bjc.6604542

**Published:** 2008-08-26

**Authors:** Davina Honess

Norman Bleehen came from an orthodox Jewish background and was much influenced by the great value both sides of his rabbinical family placed on academic achievement. He took pride in having studied and worked in internationally recognised centres of excellence at the universities of Oxford, London, Cambridge and Stanford.

Born in Manchester in 1930, he grew up in London. In 1947, he won a scholarship to Exeter College Oxford to read Medicine, taking an additional year to do a BA in Physiology and Biochemistry. A Medical Research Council (MRC) studentship allowed him to study aspects of the action of insulin in the Biochemistry Department, for which he was awarded the Gotch Memorial prize. During his years in Oxford, he was President of the Oxford University Jewish Society and was influential in the Interuniversity Jewish Federation.

In 1952, he began his clinical training at the Middlesex Hospital Medical School, qualifying in 1955. En route, he won prizes for forensic medicine, orthopaedic surgery, and radiology and radiotherapy. A house job in the radiotherapy department at the Middlesex set the direction of Norman's future career. He much impressed the then head, Professor (later Sir) Brian Windeyer. In 1957, he gained Membership of the Royal College of Physicians, before entering the Royal Army Medical Corps for his national service, initially at the British Military Hospital in Hanover and then, in 1958, in occupied Berlin. There the allied powers, Britain, the United States, France and Russia, shared responsibility, on a rotating monthly basis, for the War Criminals' gaol at Spandau. The only inmates were Baldur von Schirach, Albert Speer and, Hitler's ex-deputy, Rudolf Hess. When the British were mounting the guard, it was Norman's task to examine each inmate weekly, and to produce a report. As a committed Jew, he was deeply concerned about taking medical responsibility for these particular war criminals. Consulting his commanding officer brought the reminder: ‘This is the army, and that is your job’. But Berlin was not all duty; Norman was able to enjoy the city, especially the excellent opera.

On demobilisation in 1959, Norman returned to the Middlesex Hospital. He was to specialise in radiotherapy and gain a Fellowship of the Royal College of Radiologists. In 1966, the MRC awarded him a Lilly travelling fellowship to Stanford University, where he worked in Professor Henry Kaplan's department, much acclaimed for its excellence in relating laboratory research to treating patients. This appealed to Norman's intellect and profoundly influenced his future professional life. He thoroughly enjoyed Stanford, telling tales of never-to-be-forgotten fishing trips. Although offered a faculty post there, he chose to return to the Middlesex, where Sir Brian Windeyer had suggested that he might ultimately succeed him. Initially, the Duchess of Bedford Research Fellow, in 1969 he was appointed Professor of Radiotherapy and head of the Academic Department of Radiotherapy. He began a laboratory-based research programme alongside his clinical work and started to build the team he would subsequently take to Cambridge. That summer he met Tirza Loeb, an attractive Israeli/Australian PhD student whom he married after a whirlwind romance. They lived in a beautiful modern house in Highgate, where walls of glass gave them maximum pleasure from their garden: a growing enthusiasm. There they extended generous hospitality and friendship to Norman's rapidly expanding circle of colleagues and friends from all over the world.
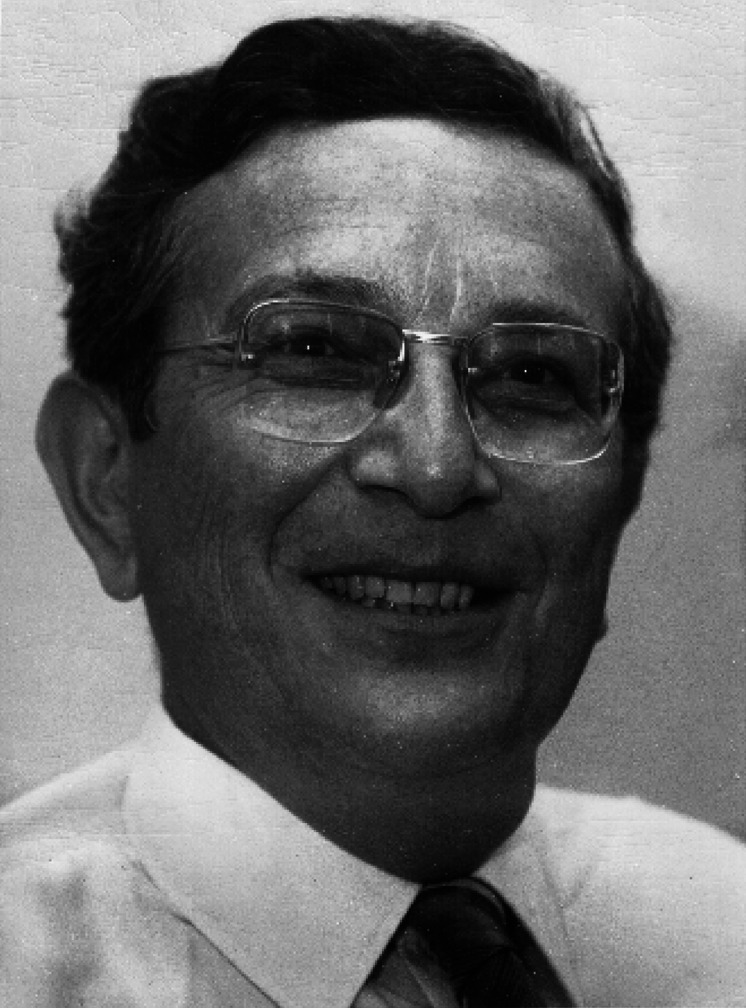


In 1975, the MRC invited Norman to set up a clinical and research unit in Cambridge at Addenbrooke's Hospital. He became Director of the MRC Clinical Oncology and Radiotherapeutics Unit (CORU). Cambridge University created a Department of Clinical Oncology endowed by the Cancer Research Campaign (now Cancer Research UK), and he was elected its first Professor of Clinical Oncology and Radiotherapeutics in the same year. Under his direction, this developed into one of the country's leading academic oncology units, both clinically and scientifically.

Clinically, his interests were the treatment of brain tumours, especially gliomas, and lung cancer, both challenging diseases where new treatments were needed to improve dismal prognoses. All novel cancer treatments must be tested for efficacy against the current best practise. At this time in the mid-1970s, national cancer clinical trials supported by the MRC were proliferating rapidly. The statistical and data management tasks associated with the good design and successful conduct of clinical trials are substantial, and as Chairman of the MRC Cancer Therapy Committee, Norman recognised the need for a dedicated MRC group to carry out these tasks. He proposed that a Cancer Trials Office (CTO) be set up within his Cambridge Unit. It was inaugurated in 1977, taking over responsibility for ongoing trials as well for as setting up new ones. The CTO was one of his major achievements; its success led the MRC to create an independent Cancer Trials Unit.

The team Norman gathered at CORU worked on both fundamental tumour biology and the development and optimisation of radiotherapy and chemotherapy. His personal research interests were in the development and trial of drugs to increase the efficacy of radiotherapy, and in the use of heat to improve radiotherapy and chemotherapy. In the course of Phase I trials of radiosensitisers and chemosensitisers, and many other clinical studies, he trained a new generation of investigative clinical oncologists and radiotherapists: now influential consultants and professors in today's oncology. The research students and post-doctoral scientists of CORU, now scattered around the world, are senior academics or hold powerful positions in the pharmaceutical industry: a potent legacy of his unit and of his leadership. Norman displayed an enviable flair for choosing people who would work together productively in a strong atmosphere of camaraderie. He was always very supportive of his staff, and invariably delivered on his promises to them, a rare quality which was much valued.

A substantial body of publications, in excess of 400, bear his name. Norman also took time to serve on numerous UK boards and committees, frequently as chairman, as well in range of international organisations, primarily in Europe. He represented the UK government in the Europe Against Cancer Programme; being passionate about smoking prevention campaigns. He served on the editorial boards of numerous cancer-related journals. He was nonetheless, a rather modest man, who did not pursue the limelight, but the worth of his work was recognised and he received many honours and awards. He was most proud of his 1990 honorary doctorate from the University of Bologna, the oldest university in the world, and his CBE for services to medicine, received in 1994, at the height of his career and influence.

Norman and Tirza were very hospitable; family, friends, colleagues and visitors, many from overseas, enjoyed their lovingly tended garden and beautiful house in quiet leafy Cambridge. An asparagus bed, particularly suited to the local soil, was Norman's special enthusiasm. Their nephews and niece have had a second home with them throughout their lives, two are continuing the medical tradition.

Norman retired in 1995, and while he remained a Fellow of St John's College and continued to serve on a variety of committees, he was able to indulge his enthusiasm for opera and his garden and to travel more extensively with Tirza, especially to her family in Australia. While there he fell ill with lung cancer, a cruel fate for a man who had spent so much of his professional career treating the disease, who had never smoked and had worked hard to deter people from smoking. Ever an academic clinician, Norman gained new insights into the disease and was gratified to find them taken up in clinical trials.

